# Attitudes Toward and Use of eHealth Technologies Among German Dermatologists: Repeated Cross-Sectional Survey in 2019 and 2021

**DOI:** 10.2196/45817

**Published:** 2024-02-12

**Authors:** Matthias Augustin, Patrick Reinders, Toni Maria Janke, Klaus Strömer, Ralph von Kiedrowski, Natalia Kirsten, Alexander Zink, Marina Otten

**Affiliations:** 1 Institute for Health Services Research in Dermatology and Nursing (IVDP) University Medical Center Hamburg-Eppendorf (UKE) Hamburg Germany; 2 Private Dermatological Practice Ahaus Germany; 3 Dermatology Specialty Practice Selters Germany; 4 Department of Dermatology and Allergy School of Medicine Technical University of Munich Munich Germany

**Keywords:** acceptance, adoption, attitude, COVID-19, dermatology, digital medicine, digitalization, pandemic, perception, teledermatology, telehealth, telemedicine

## Abstract

**Background:**

In recent years, legal and infrastructural conditions have been set to improve the adoption of digital applications in health care in Germany. The impact of these actions was amplified by the COVID-19 pandemic. So far, no studies have confirmed this progress in dermatology.

**Objective:**

The aim of this study was to measure changes in knowledge, interest, expectation, and use of digital applications in health care among dermatologists in Germany in 2019 and 2021.

**Methods:**

We administered a repeated cross-sectional survey among dermatologists in medical practices and clinics in Germany at 2 time points: t_1_ (2019; before the COVID-19 pandemic) and t_2_ (2021; during the COVID-19 pandemic). We used a standardized questionnaire, including items on respondents’ knowledge, interest, expectation, and use of digital applications, as well as their demographics. The survey was distributed by post and email. The data were analyzed descriptively as well as with multiple logistic regressions.

**Results:**

At t_1_, 585 (272/571, 47.6% female; mean age 52.4, SD 8.9 years) dermatologists and at t_2_, 792 (360/736, 48.9% female; mean age 54.3, SD 8.6 years) dermatologists participated in this survey. Interest in digital medicine was higher at t_1_ than at t_2_ (381/585, 65.1% vs 458/792, 57.8%; *P*≤.001). Nevertheless, 38.6% (306/792) had used digital applications more often since the beginning of the pandemic. For example, real-time telemedicine with patients (12/585, 2.1% vs 160/792, 7.6%; *P*≤.001) and other specialists did increase (33/385, 5.7% vs 181/792, 22.8%; *P*≤.001). Almost one-third expressed great concerns about digitalization (272/792, 34.3% vs 294/792, 37.1%; *P*=.21). Spatial analysis revealed higher interest in, more positive expectations toward, and higher use of digital applications in urban areas in comparison to rural areas. For instance, dermatologists from urban areas assessed future applications as having less risk (adjusted odds ratio [aOR] 0.51, 95% CI 0.35-0.76) than did dermatologists from rural areas. The situation was similar with the age groups, as, for example, dermatologists aged <50 years also expected lower risks (aOR 0.51, 95% CI 0.34-0.77) than those aged ≥50 years. There were no differences between sexes in use, but there were differences in knowledge and expectation; for example, male participants assessed their confidence in using digital applications as higher (aOR 1.44, 95% CI 1.01-2.04) than did female participants.

**Conclusions:**

During the pandemic, the use of digital applications in dermatology increased but still remained at a moderate level. The regional and age-related disparities identified indicate the need for further action to ensure equal access to digital care.

## Introduction

Digital health, or eHealth, can improve the effectiveness, efficiency, and equity in the delivery of health care services. The term was defined by the World Health Organization (WHO) and encompasses traditional information and communication technologies, mobile phone–based apps, artificial intelligence (AI), and computer science-driven genomics in health care [1]. Many digital health applications are nowadays established in health care environments in Europe, ranging from administrative solutions, such as practice or hospital information systems, to telemedical applications in some medical areas (eg, radiology) [2,3].

In terms of health care digitalization, Germany, along with France and Poland, is lagging behind other European countries [4]. Therefore, many legal and reimbursement conditions have been set to foster a nationwide implementation of digital health, for example, the eHealth Act in 2016 [5-8]. These initiatives enabled the reimbursement of video consultations in 2019, following the eased ban on remote treatments in 2018 [9]. Another initiative from 2019 enabled the introduction of digital health applications (Digitale Gesundheitsanwendungen; DiGAs). DiGAs are medical device-classified smartphone apps or web applications that practitioners can prescribe for patients. In terms of digital infrastructure, the nationwide telematics infrastructure (TI) is another important key element in health care digitalization in Germany. The TI aims to network all health care providers (eg, clinics, pharmacies, and outpatient practices) and thereby foster efficiency in the system by making patient data more easily available for all stakeholders [10].

Dermatology is a visual field enabling the adoption of applications such as AI, teledermatology, or patient monitoring for chronic dermatological diseases [9,11-13]. The implications of the COVID-19 pandemic rapidly amplified the adoption and use of digital applications in dermatology worldwide, but also in Germany. For example, every third German dermatologist used some form of teledermatology in May 2020, and of those, 75% had introduced it during the pandemic [14]. Yet, limited data are available on the actual uptake and acceptance of digitalization in dermatology in Germany.

Therefore, the primary aim of our survey was to measure developments in knowledge, interest, expectation, and use of digital applications among dermatologists in practices and clinics from 2019 to 2021, that is, before and after the onset of the pandemic. Our secondary aim was to analyze age, sex, and regional differences on these factors in 2021.

## Methods

### Study Design and Questionnaire

The Consensus-Based Checklist for Reporting of Survey Studies (CROSS) [15] was used for reporting this study. In June 2019 (t_1_), an anonymous, quantitative cross-sectional survey both on the web [16] and offline through a paper-based version was conducted among all German dermatologists that provided care in medical practices and clinics. The questionnaire contained items on knowledge, interest, expectation, and use of digital applications. The following demographic data were collected: the dermatologist’s age, sex, and postal code of the office or clinic. The survey was also conducted in June 2021 (t_2_), including a few additional items; the t_1_ survey had 23 items, while the t_2_ survey had 29 items (23 items were identical to t_1_ and 6 items were related to developments due to the COVID-19 pandemic, medical device–classified smartphone apps, ie, DiGAs, AI, the electronic health care professional card [elektronischer Heilberufeausweis; eHBA], and the physician’s place of work (outpatient medical practice or hospital clinic).

The majority of items were on a 5-point Likert scale. Further items with 2-4 response categories were used (eg, great importance, moderate importance, and no importance). The full questionnaires are provided in Multimedia Appendix 1. Participants had the opportunity to provide written reasons for their responses.

### Study Population, Recruitment, and Data Entry

At both time points, all members of the Federal Association of German Dermatologists (BVDD) and the German Dermatological Society (DDG) providing care in medical practices and clinics were invited to anonymously answer one of the questionnaires (on the web or offline). During the recruitment period of 4 weeks, the dermatologists received multiple reminders for participation. The total pool of addresses in both years was approximately 5900 dermatologists, but this also included members who did not practice dermatologic care anymore or had an outdated address. After considering 350 undeliverable mails or emails and an additional 30% of retirees or nonactive dermatologists in the pool, we assumed a sample of 3500 active dermatologists. No sample size calculation was conducted. To reduce errors in data entry regarding the paper-based questionnaires, 25% of all data entries were randomly checked. No errors were identified. During data cleaning, we also checked demographics for duplicates in the web-based survey–generated data.

### Statistical Analysis

Statistical analysis was performed with SPSS (version 27; IBM Corporation) for Windows, starting with descriptive statistics. For items with more than 2 answer categories, responses were binary-coded: for items with 5 answer categories, “very often” and “often” were coded as 1, while “rarely,” “never, but planned,” and “never, not planned” were coded as 2; for items with 4 answer categories, “yes and “no, but ordered” were coded as 1, while “no, not ordered” and “no, I reject a connection” were coded as 2; for items with 3 answer categories, “great importance” was coded as 1, while “moderate importance” and “no importance” were coded as 2.

Based on the postal code, participants were assigned to urban and rural regions using data from the Federal Institute for Research on Building, Urban Affairs, and Spatial Development (Bundesinstitut für Bau-, Stadt- und Raumforschung; BBSR) [17]. Age was categorized as 25-39 years, 40-49 years, 50-59 years, 60-66 years, and ≥67 years. For subgroup analysis, age was binary coded: ≥50 years and <50 years.

To test for differences between t_1_ and t_2_ (the primary aim of the study), *t* tests (2-tailed) for interval-scaled variables and chi-square tests for ordinal and nominal-scaled variables were performed (significance level of *P*=.05). Due to the exploratory nature of this research, no correction of the *P* value for multiple testing (eg, Bonferroni adjustment) was applied to prevent an increased likelihood of type II errors [18,19]. Additional adjustment for demographic and geographic differences between t_1_ and t_2_ was done through multivariate logistic regression models. Each model included age, sex, urban or rural area, and type of survey (paper or internet-based). Derived from the models, adjusted odds ratios (aORs) and the associated 95% CI are presented. Items that were introduced at t_2_ were analyzed descriptively.

To reach the secondary aim of the study, unadjusted differences between subgroups (age (≥50 vs <50 years, sex, and urban or rural) were calculated at t_2_. Additionally, aORs and 95% CI were obtained using logistic regressions.

### Missing Data

Missing data are reported for items with a rate >5%. We identified high missing values for some variables (age, sex, and region; Table 1). The Little test of missingness [20] was performed and was significant (*χ*^2^_3072_=3,704,038; *P*<.001). Hence, it was assumed that data were not missing completely at random but were missing at random [20,21]. As recommended, 20 data sets were imputed with fully conditional specification through the multiple imputation algorithm within SPSS (version 22) [22]. Thereby, linear regression was used for age, and logistic regressions were used for binary-coded variables (eg, urban or rural and sex), using all variables. After imputation, all analyses were run on each data set, and the results were pooled in accordance with the Rubin rules [23]. As there were minimal differences in the distribution of values before and after imputation, the imputed values are reported.

**Table 1:** Demographic and geographic distribution of participating dermatologists at both time points.

		t_1_ (June 2019; n=585)	t_2_ (June 2021; n=792)		*P* value
Age (years), mean (SD)		52.4 (8.9)	54.3 (8.6)		<.001^a^
**Age groups (years), n (%)**					<.001^b^
	25-39	51 (9.3)	40 (5.6)		
	40-49	139 (25.5)	162 (22.6)		
	50-59	241 (44.1)	294 (41.2)		
	60-66	95 (17.4)	181 (25.4)		
	≥67	20 (3.7)	37 (5.2)		
	Missing values	40 (6.8)	78 (9.9)		
**Sex, n (%)**					
	Female participants	272 (47.6)	360 (48.9)	.64^b^	
	Missing values	14 (2.4)	56 (7.1)		
**Activity, n (%)**					
	Activity in outpatient medical practice	N/A^c^	640 (87.9)		
	Missing values	N/A^c^	60 (7.6)		
**Region, n (%)**					
	Participants from urban areas	398 (76)	471 (76.1)	.97^b^	
	Participants from eastern federal states	112 (21.4)	130 (21)	.83^b^	
	Missing values	62 (10.6)	173 (21.8)		
Web-based participation, n (%)		284 (48.5)	261 (33)		<.001^b^

^a^2-tailed *t* tests were performed.

^b^Chi-square tests were performed.

^c^N/A: not applicable; item was first introduced in 2021.

### Ethical Considerations

For this study, no ethical review was necessary as the study was anonymous and noninterventional. The Ethics Committee of the Medical Association in Hamburg states that no ethical approval is necessary for studies in which data have already been collected anonymously (Ethik-Kommission; Sonstige Studien: Ärztekammer Hamburg [24]). The study was conducted in accordance with the principles of the Declaration of Helsinki and the Guidelines for Good Clinical Practice.

## Results

At t_1_, a total of 585 dermatologists participated, and at t_2_, a total of 792 dermatologists participated in the survey (Table 1). Considering approximately 3500 eligible dermatologists in both years, the participation rate is thereby estimated to be 16.7% (585/3500) and 22.6% (792/3500), respectively.

Sex and geographic distribution were comparable (no significant differences) between the 2 time points (Table 1), except for a mean age difference of 1.9 years between t_1_ and t_2_ (*P*≤.001). There was also a significant difference in the mode of participation: while in 2019, about 48.5% (284/585) dermatologists participated through the web-based survey, only 32.9% (261/792) did so in 2021 (*P*≤.001).

**Figure 1:**
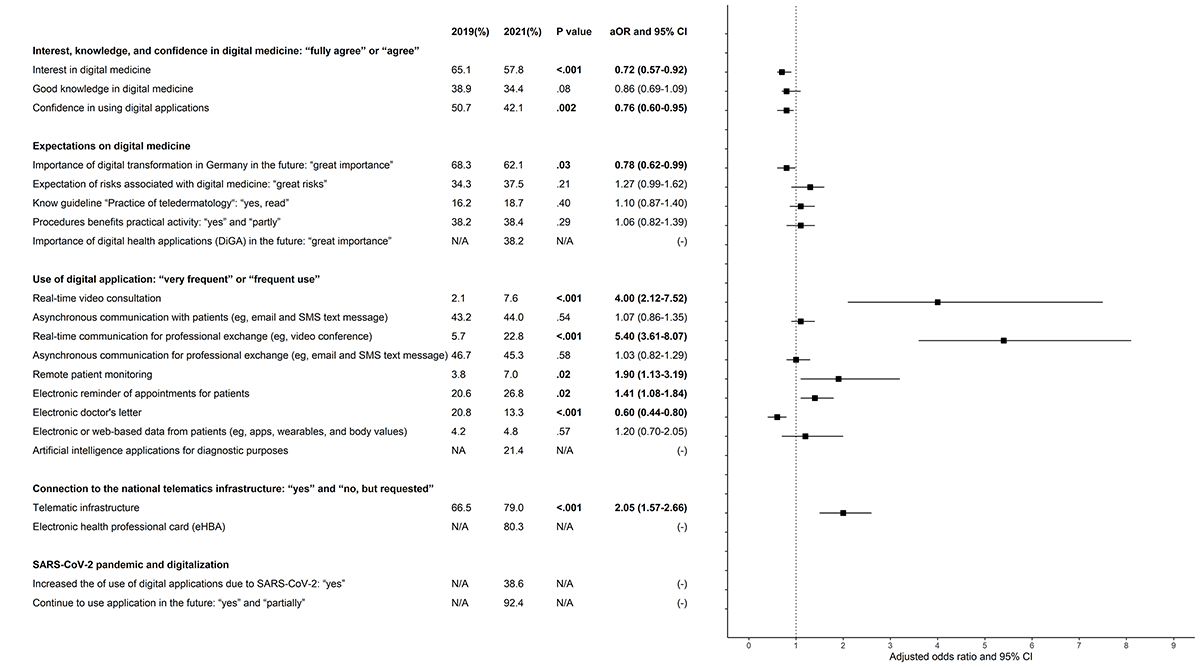
Forrest plot depicting the development of knowledge, use, and expectations of digital medicine in dermatology. Odds ratios were adjusted using logistic regressions for age groups (25-39, 40-49, 50-59, 60-66, and ≥67 years), sex (male or female), and region (urban or rural). The values in bold are significant. A chi-square test was performed to obtain *P* values. Percentages present crude or unadjusted values.

Most dermatologists expressed interest in digital medicine in both years, and half of them also felt confident in using it, but interest (381/585, 65.1% vs 458/792, 57.8%; *P*<.001) and confidence (297/585, 50.7% vs 333/792, 42.1%; *P*=.002) significantly decreased from t_1_ to t_2_ (Figure 1). The self-assessed knowledge of digital medicine was rather low in both years (228/585, 38.9% vs 272/792, 34.4%; *P*=.08)

The importance of the digital transformation within the health care system was rated highly by the participants. Yet a slight negative trend was noticed (t_1_: 400/585, 68.3% vs t_2_: 492/792, 62.1%; *P*=.03) (Figure 1). For around one-third of participants, digital medicine harbors a high risk (t_1_: 201/585, 34.3% vs t_2_: 294/792, 37.1%; *P*=.21) and supports daily activities (223/585, 38.2% vs 304/792, 38.4%; *P*=.93). Only a small number of dermatologists had read the guideline on teledermatology (t_1_: 95/585, 16.2% vs t_2_: 148/792, 18.7%; *P*=.40). DiGAs were seen as important in the future by 38.2% (303/792) of participants at t_2_.

The most often used digital applications in dermatology at both survey time points were those ensuring asynchronous communication (store-and-forward) with patients (t_1_: 253/585, 43.2% and t_2_: 348/792, 44%; *P*=.54) and other specialists (t_1_: 273/585, 46.7% and t_2_: 359/792, 45.3%; *P*=.58). Real-time video consultation with a patient was used 4 times more frequently in 2021 compared with 2019 but was still used rarely (t_1_: 12/585, 2.1%, and t_2_: 60/792, 7.6%; aOR 4.00, 95% CI 2.12-7.52) (Figure 1). Real-time communication with other specialists was used nearly 6 times more often (t_1_: 33/585, 5.7%, and t_2_: 181/792, 22.8%; *P*<.001). Remote patient monitoring (22/585, 3.8% vs 55/792, 7%; *P*=.02) and electronic appointment reminders (t_1_: 121/585, 20.6% vs t_2_: 212/792, 26.8%; *P*=.02) were also used more regularly by dermatologists in 2021. In contrast, electronic physician letters were sent significantly less often in 2021 compared with 2019 (t_1_: 122/585, 20.8% vs t_2_: 105/792, 13.3%; *P*<.001). Incorporation of web-based patient data into patient care was rarely used at both time points (t_1_: 25/585, 4.2% vs 38/792, 4.8%; *P*=.57) (Figure 1).

Connection to the nationwide TI has progressed further, with 79% (626/792) of participants being connected or having requested a connection in comparison to 66.5% (389/792) in 2019 (*P*<.001). The eHBA was available or at least requested by 80.3% (636/792), and AI methods for diagnosing were used by 21.4% (169/792) of participating dermatologists at t_2_. For the last 2 items, no data were available for t_1_.

According to respondents, 38.6% (306/792) had used digital medicine procedures more frequently since the onset of the COVID-19 pandemic. Of these, 92.4% (283/306) estimated to make at least partial continued use of the newly introduced digital applications.

Younger dermatologists indicated higher interest, knowledge, and confidence in using digital applications (aORs in Table 2). They saw more of a benefit in using applications for their daily activities than older dermatologists. Younger participants also used asynchronous communication methods with patients and physicians and electronic appointment reminders for patients more often, whereas older dermatologists more often expected high risks regarding the implementation of digital medicine.

Participants practicing in urban areas had a higher likelihood of reporting a good level of knowledge, interest, and confidence in using digital applications (aORs in Table 3). Furthermore, they used many of the digital applications more often, including asynchronous communication with patients and colleagues and electronic patient reminders, and rated the risks associated with digital medicine as lower. In contrast, they were less likely connected to the TI.

Only a few significant differences in sexes were identified (aORs in Table 4). Male dermatologists were more likely than female dermatologists to state a good level of knowledge and confidence in using digital applications. Female dermatologists more often expected the great importance of DiGAs and the digital transformation in the future.

**Table 2:** Adjusted odds ratios (aORs) for associations between age groups (≥50 years vs <50 years) and knowledge, interest, expectation, and use of digital applications in dermatology (survey date: 2021). The values in italics are significant.

		Age ≥50 years (n=566^a^), n (%)^b^	Age <50 years (n=223^a^), n (%)^b^	*P* value^c,b^	aOR (95% CI) for age ≥50/< 50 years; reference ≥50 years^d^
**Interest, knowledge, and confidence in digital medicine: “fully agree” or “agree”**					
	Interest in digital medicine	*304 (53.7)*	*158 (70.9)*	*<.001*	*2.15 (1.47-3.15)*
	Good knowledge of digital medicine	*180 (31.8)*	*96 (43.0)*	*<.001*	*1.78 (1.25-2.54)*
	Confidence in using digital applications	*220 (38.9)*	*118 (52.9)*	*<.001*	*1.91 (1.33-2.72)*
**Use of digital application: “very frequent” or “frequent”**					
	Real-time video consultation	40 (7.1)	22 (9.9)	.24	1.29 (0.68-2.46)
	Asynchronous communication with patients (eg, email and SMS text message)	*235 (41.5)*	*121 (54.3)*	*<.001*	*1.65 (1.16-2.34)*
	Real-time communication for professional exchange (eg, video conference)	*118 (20.8)*	*66 (29.6)*	*<.001*	1.45 (0.98-2.47)
	Asynchronous communication for professional exchange (eg, email and SMS text message)	*237 (41.9)*	*127 (57.0)*	*<.001*	*1.73 (1.23-2.44)*
	Remote patient monitoring	36 (6.4)	20 (9.0)	.22	1.47 (0.72-2.99)
	Electronic reminder of appointments for patients	*140 (24.7)*	*77 (34.5)*	*.005*	*1.63 (1.11-2.38)*
	Electronic doctor’s letter	77 (13.6)	32 (14.3)	.62	0.97 (0.56-1.67)
	Electronic or web-based data from patients (eg, apps, wearables, and body values)	26 (4.6)	13 (5.8)	.28	1.63 (0.70-3.83)
	Artificial intelligence applications for diagnostic purposes	115 (20.3)	52 (23.3)	.35	1.07 (0.72-1.60)
**Expectations of digital medicine**					
	Importance of digital transformation in Germany in the future: “great importance”	*341 (60.2)*	*159 (71.3)*	*.007*	1.43 (0.98-2.10)
	Expectation of risks associated with digital medicine: “great risks”	*247 (42.6)*	*58 (26.0)*	*<.001*	*0.51 (0.34-0.77)*
	Know guideline “Practice of teledermatology:” “yes, read”	117 (20.7)	34 (15.2)	.10	0.85 (0.51-1.40)
	Procedures benefits practical activity: “yes” and “partly”	*222 (39.2)*	*118 (52.9)*	*<.001*	*1.68 (1.19-2.39)*
	Importance of digital health applications (DiGA^e^) in the future: “great importance”	211 (37.3)	90 (40.4)	.43	0.97 (0.68-1.38)
**Connection to the national telematics infrastructure: “yes” and “no, but requested”**					
	Telematic infrastructure	473 (83.6)	173 (77.6)	.08	0.75 (0.47-1.18)
	Electronic health professional card (eHBA^f^)	461 (81.4)	171 (76.7)	.09	0.82 (0.52-1.31)
**SARS-CoV-2 pandemic and digitalization**					
	Increased the use of digital applications due to SARS-CoV-2: “yes”	*184 (32.5)*	*111 (49.8)*	*<.001*	*1.85 (1.26-2.71)*
	Continue to use application in the future: “yes” and “partially”	173 (94.0)	104 (93.7)	.78	0.91 (0.28-2.93)

^a^Does not add up to the overall total of 792, as data for an average of 3 participants were not imputed.

^b^Unadjusted or crude values.

^c^Chi-square tests were performed.

^d^Logistic regressions were performed. Following items were included as independent variables: sex (male or female) and regional allocation (urban or rural).

^e^DiGAs: Digitale Gesundheitsanwendungen.

^f^eHBA: elektronischer Heilberufeausweis.

**Table 3:** Adjusted odds ratios (aORs) for associations between region (urban or rural) and knowledge, interest, expectation, and use of digital applications in dermatology (survey date: 2021). The values in italics are significant.

			Urban (n=600), n (%)^a^		Rural (n=192), n (%)^a^		*P* value^b^		aOR (95% CI) for urban/rural; reference: rural^c^
**Interest, knowledge, and confidence in digital medicine: “fully agree” or “agree”**									
	Interest in digital medicine	*372 (62)*		*91 (47.4)*		*<.001*		*1.70 (1.17-2.47)*	
	Good knowledge of digital medicine	*228 (38)*		*50 (26)*		*.01*		*1.64 (1.06-2.49)*	
	Confidence in using digital applications	*279 (46.5)*		*61 (31.8)*		*.002*		*1.73 (1.17-2.58)*	
**Use of digital application: “very frequent” or “frequent”**									
	Real-time video consultation	55 (9.2)		7 (3.6)		.05		2.68 (0.95-7.51)	
	Asynchronous communication with patients (eg, email and SMS text message)	*295 (49.2)*		*63 (32.8)*		*.01*		*1.79 (1.20-2.68)*	
	Real-time communication for professional exchange (eg, video conference)	*154 (25.7)*		*30 (15.6)*		*.02*		*1.90 (1.13-3.17)*	
	Asynchronous communication for professional exchange (eg, email and SMS text message)	*297 (49.5)*		*70 (36.5)*		*<.001*		*1.62 (1.10-2.39)*	
	Remote patient monitoring	45 (7.5)		12 (6.3)		.58		1.19 (0.56-2.52)	
	Electronic reminder of appointments for patients	*185 (30.8)*		*33 (17.2)*		*<.001*		*2.01 (1.24-3.27)*	
	Electronic doctor’s letter	89 (14.8)		20 (10.4)		.22		1.54 (0.83-2.86)	
	Electronic or web-based data from patients (eg, apps, wearables, and body values)	32 (5.3)		8 (4.2)		.51		1.31 (0.49-3.56)	
	Artificial intelligence applications for diagnostic purposes	132 (22.0)		38 (19.8)		.52		1.12 (0.70-1.82)	
**Expectations of digital medicine**									
	Importance of digital transformation in Germany in the future: “great importance”	*400 (66.7)*		*104 (54.2)*		*<.001*		*1.64 (1.08-2.48)*	
	Expectation of risks associated with digital medicine: “great risks”	*205 (34.2)*		*101 (52.6)*		*<.001*		*0.51 (0.35-0.76)*	
	Know guideline “Practice of teledermatology:” “yes, read”	84 (14.0)		25 (13.0)		.79		1.23 (0.74-2.08)	
	Procedures facilitate practical activity: “yes” and “partly”	270 (45.0)		72 (37.5)		.10		1.28 (0.86-1.93)	
	Importance of digital health applications (DiGA^d^) in the future: “great importance”	185 (39.8)		48 (33.3)		.14		1.30 (0.87-1.95)	
**Connection to the national telematics infrastructure: “yes” and “no, but requested”**									
	Telematic infrastructure	*473 (78.8)*		*175 (91.1)*		*<.001*		*0.37 (0.21-0.68)*	
	Electronic health professional card (eHBA^e^)	471 (78.5)		163 (84.9)		.06		0.61 (0.35-1.31)	
**SARS-CoV-2 pandemic and digitalization**									
	Increased the use of digital applications due to SARS-CoV-2: “yes”	*237 (39.5)*		*59 (30.7)*		*.05*		1.37 (0.91-2.06)	
	Continue to use application in the future: “yes” and “partially”	221 (93.2)		57 (96.6)		.50		0.63 (0.14-2.95)	

^a^Unadjusted or crude values.

^b^Chi-square tests were performed.

^c^Logistic regressions were performed. Following items were included as independent variables: age (≥50 years or < 50 years) and sex (male or female).

^d^DiGAs: Digitale Gesundheitsanwendungen.

^e^eHBA: elektronischer Heilberufeausweis.

**Table 4:** Adjusted odds ratios (aORs) for associations between sex and knowledge, interest, expectation, and use of digital applications in dermatology (survey date: 2021). The values in italics are significant.

		Female (n=388), n (%)^a^	Male (n=404), n (%)^a^	*P* value^b^	aOR (95% CI) for male/female; reference: female^c^
**Interest, knowledge, and confidence in digital medicine: “fully agree” or “agree”**					
	Interest in digital medicine	224 (57.7)	240 (59.4)	.94	1.22 (0.87-1.74)
	Good knowledge of digital medicine	*119 (30.7)*	*158 (39.1)*	*.02*	*1.71 (1.18-2.46)*
	Confidence in using digital applications	154 (39.7)	186 (46.0)	.09	*1.44 (1.01-2.04)*
**Use of digital application: “very frequent” or “frequent”**					
	Real-time video consultation	31 (8.0)	31 (7.7)	.86	1.01 (0.52-1.97)
	Asynchronous communication with patients (eg, email and SMS text message)	174 (44.8)	184 (45.5)	.86	1.19 (0.84-1.67)
	Real-time communication for professional exchange (eg, video conference)	100 (25.8)	85 (21.0)	.10	0.79 (0.53-1.19)
	Asynchronous communication for professional exchange (eg, email and SMS text message)	187 (48.2)	179 (44.3)	.24	0.92 (0.65-1.29)
	Remote patient monitoring	30 (7.7)	27 (5.7)	.45	0.94 (0.48-1.86)
	Electronic reminder of appointments for patients	98 (25.3)	119 (29.5)	.34	1.35 (0.92-1.98)
	Electronic doctor’s letter	53 (13.7)	56 (13.7)	.89	0.79 (0.48-1.31)
	Electronic or web-based data from patients (eg, apps, wearables, and body values)	16 (4.1)	23 (5.7)	.30	1.57 (0.69-3.57)
	Artificial intelligence applications for diagnostic purposes	93 (24.0)	77 (19.1)	.09	0.87 (0.57-1.31)
**Expectations of digital medicine**					
	Importance of digital transformation in Germany in the future: “great importance”	*267 (68.8)*	*237 (58.7)*	*.04*	*0.67 (0.49-0.93)*
	Expectation of risks associated with digital medicine: “great risks”	*132 (34.0)*	*173 (42.8)*	*.02*	1.39 (0.97-1.97)
	Know guideline “Practice of teledermatology:” “yes, read”	58 (14.9)	79 (19.6)	.09	1.32 (0.85-2.05)
	Procedures eases practical activity: “yes” and “partly”	175 (45.1)	167 (41.3)	.34	1.13 (0.80-1.60)
	Importance of digital health applications (DiGA^d^) in the future: “great importance”	*167 (43.0)*	*134 (33.2)*	*.05*	*0.57 (0.40-0.80)*
**Connection to the national telematics infrastructure: “yes” and “no, but requested”**					
	Telematic infrastructure	322 (83.0)	326 (80.7)	.43	0.96 (0.63-1.47)
	Electronic health professional card **(**eHBA^e^)	314 (80.9)	320 (79.2)	.41	0.97 (0.64-1.49)
**SARS-CoV-2 pandemic and digitalization**					
	Increased the use of digital applications due to SARS-CoV-2: “yes”	*162 (41.8)*	*135 (33.4)*	*.02*	0.82 (0.32-2.10)
	Continue to use applications in the future: “yes” and “partially”	151 (93.2)	127 (94.1)	.50	0.40 (0.13-1.28)

^a^Unadusted or crude values.

^b^Chi-square tests were performed.

^c^Logistic regressions were performed. The following items were included as independent variables: age (≥50 years or <50 years) and regional allocation (urban or rural).

^d^DiGAs: Digitale Gesundheitsanwendungen.

^e^eHBA: elektronischer Heilberufeausweis.

## Discussion

### Overview

This trend analysis is the first to measure developments in knowledge, interest, expectation, and use of digital applications in dermatology during the events of the COVID-19 pandemic. First, we identified a moderate increase in the use rate of digital applications in dermatological care, while interest in the topic decreased slightly; one-third of practitioners expect great risks with the introduction of digital medicine. Second, we noticed regional and age-related disparities in self-assessed knowledge, confidence, expectations of digital medicine, and the use rate of digital applications. The survey also revealed differences between sexes in self-assessed knowledge and confidence in digital medicine among dermatologists in Germany.

The decline in interest in using digital applications might be explained by the more widespread implementation of digital applications, as verified by this survey. We hypothesize that digital applications may have moved from extraordinary to ordinary daily routines, resulting in a reduction of interest. Confidence and knowledge may have declined as practitioners were more often confronted with the actual use of applications in their daily routine, potentially disclosing the limits of their knowledge in digital medicine.

Interestingly, only one-third of physicians stated that digital applications support their daily activities, which is one of the main promises of health care digitalization. The outcome could be caused by the long, costly, and not yet fully implemented nationwide eHealth initiatives in Germany [25]. The overall perception will, however, differ depending on the type of digital application. The benefit of AI for diagnostic purposes, for example, might be high, as the technology could potentially improve the efficiency of screening and the diagnostic confidence of practitioners [26].

We observed an increase in real-time communication technologies to communicate with patients and other physicians, which could also be shown by other studies [14]. Compared to the United States, where nearly 42.6% of offices stated using video consultations, this service is rarely offered by dermatologists in Germany, as identified within our results (160/792, 7.6%) [27]. Reasons for the low use may involve insufficient reimbursement for providers and a 2019-loosened ban on remote treatments, which also explains the low use rate for this year [14]. In the future, it can be assumed that video consultation with patients may decline after the pandemic fades into the background again, as the main reason for its use was protection from infections [2]. Lower use of video consultations in times of low COVID-19 infection rates could already be shown in Germany [28]. Nevertheless, it is not assumed that the use rates of video consultation will go back to the use rates of the prepandemic era.

On the contrary, the use of asynchronous communication technologies was not affected by the pandemic or other changes during the observed period, which contradicts other results from Elsner [14] at first glance, wherein 75% of dermatologists in 2020 did introduce the technology with the onset of the pandemic. Within our survey, all purposes—administrative (eg, appointments and invoices), medical-related (eg, diagnosis), and technologies (eg, email, SMS text messages, or teledermatological platforms)—were covered by the item, whereas the item in the survey by Elsner [14] was specific to store-and-forward teledermatology, so a medical-related purpose. As the number of dermatologists adopting store-and-forward teledermatology substantially increased, we assume that the purpose of asynchronous communications has partly shifted from administrative purposes to medical-related purposes with the onset of the pandemic.

In terms of the TI, dermatologists are a bit behind other specialist groups: 14% of all practitioners that responded to a nationwide survey [2] and 21% (166/792) of dermatologists within our survey are not yet connected to the TI. Regarding DiGAs, they see a similar importance to other physicians: 40% nationwide of all physicians versus 38.2% (303/792) in our survey. We found a reduction in use of the electronic physician’s letter in dermatology, although most outpatient physicians stated that it eases the administrative workload in clinical practice [2].

Within the survey, age, sex, and regional differences were identified. The lower self-assessed knowledge and confidence in digital medicine by female dermatologists could be confounded by social expectations. In other areas of life, no or only marginal differences in digital literacy between sexes could be measured when accounting for education and employment status [29]. Nevertheless, female dermatologists could thus have similar knowledge of digital medicine, resulting in similar use rates, as shown.

The phenomenon of regional and age disparities, noted in our survey, has generally been noted with the advent of digitalization in all areas of life and is known as the digital divide. Thus, not only access to the internet, internet speed, and availability of appropriate hardware, but also digital competencies among practitioners and patients are related to inequalities in the use of digital tools [30]. In Germany, the average use rates of the internet, as well as digital competence and openness to use digital applications, are more pronounced in urban regions and younger age groups in comparison to rural regions and older groups [31], partially explaining the differences identified within our survey. Consequently, digital applications have the potential to increase unequal access to care, at least in the short term. To ensure equity in the delivery of care in dermatology, all disparities related to sex, age, or region should be addressed by legal, infrastructure, and reimbursement initiatives. In addition, implementation strategies for evidence-based digital applications should be customized to specific target groups [32].

The response rates of our surveys were slightly lower than in previous surveys among German dermatologists [33]. Hence, a certain selection bias among participants cannot be excluded. Considering the official physician statistics, dermatologists aged between 50 and 59 years were slightly overrepresented, whereas dermatologists aged 35 years or younger were slightly underrepresented. In addition, female dermatologists were underrepresented in the survey in comparison to the federal statistic (360/736, 48% in our survey vs 60% at the national level) [34]. Although our surveys were not representative, the sample sizes were large enough to detect trends and variations among demographic and geographic parameters. To improve comparability between survey waves, an adjustment of demographic and geographic differences was performed through multiple logistic regressions. Groups at t_1_ and t_2_ may differ for unmeasured characteristics (eg, professional expertise, number of consultations per week, socioeconomic patient groups in the area, and the number of private and statutory insured patients), thus resulting in underestimated or overestimated differences. Nevertheless, the same population was contacted at both time points, and important baseline characteristics did not present any larger differences.

### Conclusions

The repeated survey revealed an increase in the adoption of the majority of digital applications in dermatology, amplified by the COVID-19 pandemic. However, regional and age-related disparities in the use of digital applications exist, reducing equal access to innovative health care solutions for patients. In the future, policy strategies must be developed to counteract these disparities and improve nationwide implementation. Developers of digital innovations should incorporate physicians’ perspectives more commonly to ensure feasibility and use. Future research should support policymakers and developers by identifying barriers to implementation, monitoring disparities, and ensuring an evidence base for digital applications. Widespread implementation and acceptance among patients and physicians can then improve the efficiency, equity, and effectiveness of care.

## Appendix

Multimedia Appendix 1 Survey: Digital medicine in German dermatology practices and clinics 2019 and 2021.

## Abbreviations

AI: artificial intelligence
BBSR: Bundesinstitut für Bau-, Stadt- und Raumforschung
BVDD: Federal Association of German Dermatologists
CROSS: Consensus-Based Checklist for Reporting of Survey Studies
DDG: German Dermatological Society
DiGAs: Digitale Gesundheitsanwendungen
eHBA: elektronischer Heilberufeausweis
TI: telematics infrastructure
WHO: World Health Organization

## Data Availability

The data sets generated during and/or analyzed during this study are available from the corresponding author on reasonable request.
