# Supplementary material for: Attitudes Toward and Use of eHealth Technologies Among German Dermatologists: Repeated Cross-Sectional Survey in 2019 and 2021
Source: J Med Internet Res. 2024 Feb 12;26:e45817. doi: 10.2196/45817 (PMC10897787; doi:10.2196/45817)
Supplement: Multimedia Appendix 1 [file jmir_v26i1e45817_app1.docx]

**Multimedia Appendix 1**. Survey: Digital medicine in German dermatology practices and clinics 2019 and 2021.

**Survey: Digital medicine in German Dermatological Practices in 2019**

1. **General characteristics:**

Postcode of practice: ___________

Number of dermatologists in your practice: ___________

Sex: *male / female*

Age: ____________

1. **To what extent do you agree with the following statements?**

*(Likert scale: Fully agree; agree; undecided; disagree; fully disagree)*

- I am personally interested in the topic of digital medicine.
- I have good knowledge of digital medicine.
- I feel safe using digital/online applications.

1. **Which digital medicine procedures do you use in your practice?**

*(Likert scale: Very often; often; seldom; never, but planned; never, not planned)*

- Real-time video consultation
- Asynchronous communication with patients (eg, email and SMS text message)
- Real-time communication for professional exchange
- Asynchronous communication for professional exchange (eg, email and SMS text messages)
- Remote patient monitoring
- Electronic reminder of appointments for patients
- Electronic doctor’s letter
- Electronic or web-based data from patients (eg, apps, wearables, and body values)
- Other: ________________

1. **Have you already connected your practice to the telematics infrastructure (TI)?**

*Answer: Yes; No, but ordered; No, not ordered yet; No, I reject a connection*

1. **What proportion of your patients do you treat using telemedical procedures?**

**approx. ___ %**

1. **Do these procedures benefit your practical activities?** *Answer: yes; partly; no*
2. **Do you store your patients' data digitally?** *Answer: yes, exclusively; yes, partly, no*
3. **In your opinion, what importance will the digital transformation have in Germany in the future?** *Answer: no importance, medium importance, great importance*
4. **Do you expect risks of digital medicine in dermatology?** *Answer:* *no risks, medium risks, great risks*
5. **Are you aware of the guideline on teledermatology?** *Answer: Yes, read; yes, aware; no*

**Survey: Digital medicine in German Dermatological Practices and Clinics in 2021**

1. **General characteristics:**

Postcode of practice: ___________

Activity in: *medical practice; outpatient clinic*

Number of dermatologists in your practice: ___________

Sex: *male / female*

1. **To what extent do you agree with the following statements?**

*(Likert scale: Fully agree; agree; undecided; disagree; fully disagree)*

- I am personally interested in the topic of digital medicine.
- I have good knowledge of digital medicine.
- I feel safe using digital/online applications.

1. **Which digital medicine procedures do you use in your practice?**

*(Likert scale: Very often; often; seldom; never, but planned; never, not planned)*

- Real-time video consultation
- Asynchronous communication with patients (eg, email and SMS text message)
- Real-time communication for professional exchange
- Asynchronous communication for professional exchange (eg, email and SMS text messages)
- Remote patient monitoring
- Electronic reminder of appointments for patients
- Electronic doctor’s letter
- Electronic or web-based data from patients (eg, apps, wearables, and body values)
- AI applications for diagnostic purposes
- Other: ____________________

1. **Have you already connected your practice to the telematics infrastructure (TI)?**

*Answer: Yes; No, but ordered; No, not ordered yet; No, I reject a connection*

1. **Do you already have an electronic health professional card (eHBA)?**

*Answer: Yes; No, but ordered; No, not ordered yet; No, I reject a connection*

1. **What proportion of your patients do you treat using telemedical procedures?
   approx. ___ %**
2. **Do these procedures benefit your practical activities?** *Answer: yes; partly; no*
3. **Do you store your patients' data digitally?** *Answer: yes, exclusively; yes, partly, no*
4. **In your opinion, what importance will the digital transformation have in Germany in the future?** *Answer: no importance, medium importance, great importance*
5. **Do you expect risks of digital medicine in dermatology?** Answer: *no risks, medium risks, great risks*
6. **How important do you think digital health applications (DiGAs) will be in the future?** *Answer: no importance, medium importance, great importance*
7. **Are you aware of the guideline on teledermatology?** *Answer: Yes, read; yes, aware; no*
8. **Since the start of the corona pandemic, have you been using digital medicine procedures more commonly?** *Answer: yes; no*
9. **Will you continue to use these interventions in the future?** *Anwer: yes; no*
